# Proactive Career Orientation and Subjective Career Success: A Perspective of Career Construction Theory

**DOI:** 10.3390/bs13060503

**Published:** 2023-06-15

**Authors:** Po-Chien Chang, Yuanli Guo, Qihai Cai, Hongchi Guo

**Affiliations:** 1 School of Business, Macau University of Science and Technology, Macau 999078, China; 2 Beidahuang Group Co., Ltd., Harbin 150036, China

**Keywords:** career construction theory, proactive career orientation, career adaptability, mentoring, subjective career success

## Abstract

In the current dynamic and flexible work environment, traditional career models are constantly challenged by individuals’ self-concepts of career development. Previous studies have investigated the predictors of subjective career success, yet little is known about the impact of proactive career orientation on subjective career success. This study, grounded in the career construction theory, aims to examine the influence mechanism of proactive career orientation on subjective career success by analyzing questionnaire data from 296 employees. Empirical results indicate that proactive career orientation has a positive impact on subjective career success. Second, career adaptability partially mediates the relationship between proactive career orientation and subjective career success. Third, mentoring moderates the relationship between proactive career orientation and career adaptability, as well as the relationship between career adaptability and subjective career success. Specifically, both the positive impact of proactive career orientation on career adaptability and the positive impact of career adaptability on subjective career success are stronger when the level of mentoring is higher. Fourth, the indirect relationship between proactive career orientation and subjective career success through career adaptability is stronger when mentoring is high compared to when mentoring is low. This study contributes to the career construction theory by identifying the influence mechanism of proactive career orientation on subjective career success through career adaptability, with mentoring moderating the process. As for practical implications, research findings remind managers of the importance of career planning and mentorship in enhancing employees’ subjective career successes.

## 1. Introduction

In the VUCA (volatility, uncertainty, complexity, and ambiguity) era, there are many opportunities for individual career development but also more challenges and pressures. Understanding careers is important to individuals, organizations, and society [1]. How to achieve career success has become a heated topic, attracting scholarly attention. With the dynamic organizational environment and the development of boundaryless careers, individuals are more concerned with subjective feelings at work and career management, and assess career success according to their personal criteria, needs, values, career stages and aspirations [2]. Traditional career success indicators to measure objective career success, such as salary and promotion, have become less reflective of employee career satisfaction. More attention has been shifted to subjective career success, which refers to individuals’ perception and experience of achieving personally meaningful career outcomes [3,4]. Spurk et al. (2019) demonstrated that subjective career success not only negatively predicted withdrawal outcomes (e.g., turnover intentions and actual retirement) but also positively affect career attitude outcomes and well-being [5]. Subjective career success, in this regard, has a significant role both for the individual and the organization. Thus, career construction theory, which characterizes adaptation outcomes as resulting from adaptive readiness, adaptability resources and adapting responses [6], provides us with important insights to understand how to achieve career success more effectively.

The existing literature has examined the predictors of individuals’ subjective career success [7,8,9]. However, with careers becoming less predictable in the current dynamic and flexible work environment, employees are more responsible for their career development [10]. Studies on how contemporary career orientation affects subjective career success seem to be more necessary [11,12]. Proactive career orientation, reflecting a general focus on personally driven, goal-directed work behavior and including self-directed, value-driven and psychological mobility constructs as components [13], is the driver of the way individuals enact and evaluate careers in contemporary organizations [14]. Yet, studies on the effects of proactive career orientation on subjective career success are scarce. Studies exploring proactive career orientations and how they contribute to enhanced career outcomes are needed [15]. To that end, consistent with career construction theory [6], we conceptualize proactive career orientation as an adaptive readiness and aim to investigate the potential positive effect of proactive career orientation on subjective career success.

Career construction theory explains the dynamic process wherein individuals build their career development through a series of meaningful vocational behaviors and work experiences [6]. Considered as a self-regulating psychosocial ability, career adaptability is a core element of this process, helping individuals develop adaptive strategies and behaviors to achieve adaptive goals for better career development outcomes [16]. Proactive career orientations that focus on self-directed, value-driven, and psychological mobility in a protean and boundaryless career attitude, have been shown to significantly predict career adaptability [17,18]. Furthermore, career adaptability helps employees to successfully manage their career development and adjustment, thereby enhancing subjective career success [8]. We, therefore, argue that career adaptability may mediate the relationship between proactive career orientation and subjective career success.

The career construction theory states that individual career development is influenced by the interaction of individuals and situations [6], providing insight into the potential boundary conditions in the above mechanism. Currently, there are fewer studies exploring situational factors in the career construction process, while it is very critical [19]. Mentoring, as an important workplace factor, is a set of role activities, including coaching, role modeling and social support that mentors provide to proteges [20,21], which contributes to the mentee’s career adaptability and subjective career success [22]. For example, empirical studies find that mentoring can facilitate a mentee’s personal learning, skill development and career adaptability [23]. Similarly, a meta-analysis of mentoring demonstrates that mentoring helps mentees access to career success, especially subjective career success, by providing them with vocational support, psychosocial support, and role modeling support [24]. Hence, we expect mentoring to function as a potential moderator of the relationships between proactive career orientation, career adaptability and subjective career success.

**Figure 1:**
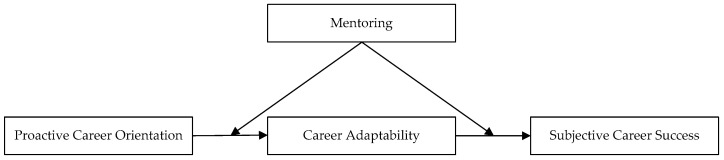
Proposed moderated mediation model.

Building on career construction theory [6], we thus propose a moderated mediation model, which jointly examines career adaptability as the mediating mechanism, and mentoring as the moderator of the relationships between proactive career orientation, career adaptability and subjective career success (see Figure 1). This study contributes to the existing literature in the following four aspects. First, we contribute to the literature on subjective career success by showing that proactive career orientation positively predicts subjective career success. Our research answers calls for efforts to promote practitioners’ subjective career success [5]. Second, our work contributes to extending the application of career construction theory. We conceptualize proactive career orientation as an adaptive readiness and career adaptability as an adaptability resource, indicating how proactive career orientation via career adaptability improves subjective career. Third, this study clarifies mentoring as a boundary condition to enhance the relationship between proactive career orientation and career adaptability, as well as the relationship between career adaptability and subjective career success, answering the call of Hirschi and Koen (2021) to unpack the role of context in the influence process of contemporary career orientation on career self-management [25]. Finally, we built a career success path for practitioners available within educational institutions or companies, which may benefit both employers and employees [26].

## 2. Literature Review and Hypotheses

### 2.1. Proactive Career Orientation and Subjective Career Success

According to the career construction theory, individual adaptive readiness is an important antecedent to obtaining adaptive resources or outcomes, and the process of adaptation is facilitated and directed by one’s goals, which harmonize inner needs with outer opportunities [6,16]. Proactive career orientation is an individual’s subjective adaptation intention, which can help individuals focus on their own opportunities in career development and attain career success by seizing these opportunities [27]. Employees with proactive career orientation are self-directed, which means they can make their own decisions and possess a positive attitude in career development (such as active goal setting, seeking career opportunities, promoting skills, etc.), rather than waiting or relying on others [28]. People who are self-directed are more susceptible to environmental changes and adjustments to their career development, which can contribute to a greater sense of self-determination, thereby increasing their career satisfaction [14,29]. Moreover, individuals with proactive career orientation are also value-driven, adjusting their career development according to their values, motivations and needs [30,31]. Proactive career orientation allows employees to pursue what they believe to be meaningful based on their own values, without caring too much about what others think, which facilitates subjective career success [28]. Similarly, employees with proactive career orientation view challenges at work as career development opportunities, which help them gain more career experiences to improve their competitiveness and ultimately promote career success [32]. On the other hand, individuals with proactive career orientation tend to have high levels of career self-awareness and well-developed career identities, as well as believing that they can express themselves authentically in their careers [13]. This makes them more likely to feel satisfied with their careers. Proactivity in career orientation helps individuals to use career self-management as a way to gain access to more positive career outcomes [25]. So, we propose the following hypothesis:

**Hypothesis 1.**  *Proactive career orientation is positively associated with subjective career success*.

### 2.2. Mediating Role of Career Adaptability

According to career construction theory, an individual’s adaptive readiness affects his or her career adaptability, which in turn affects adaptive behavior and adaptive outcomes [16]. Subjective career success, as an adaptation outcome, reflects the individual is in a state of adaptation or satisfaction in his or her career [33]. The process of achieving subjective career success is a self-constructed career adaptation process that begins with the individual’s adaptation willingness. Such a willingness is the intrinsic motivation for career adaptability, which can be expressed in personality, career orientation, preferences, and so on [17,34]. As a career orientation, proactive career orientation represents the individual’s adaptive willingness. Employees with a proactive career orientation are independent of the organization and in charge of their own careers, which motivates them to develop their skills through self-directed learning and to seek external resources and support to enhance career adaptability [35]. Moreover, employees with a proactive career orientation are more optimistic about coping with changes in the environment, such as adjusting their goals flexibly, viewing changes as opportunities, and improving their career management skills so that they are adaptable to changes and challenges [31]. Consequently, proactive career orientation has a positive impact on career adaptability.

Additionally, career construction theory proposes that individuals with higher career adaptability will perform more adaptive behaviors and outcomes. Career adaptability reflects the psychosocial capital of how people adapt to the interaction of career and environment, which facilitates their career concern, curiosity, control, and confidence [16]. Specifically, career concern disposes employees to focus on changes and turn challenges into opportunities [36]; career control emphasizes a behavioral competency that employees are able to shape their careers by proactively taking action [37]; career curiosity motivates employees to actively try and explore at work [38]; and career confidence sustains employees’ belief to cope with difficulties and achieve career success, all of these contribute to satisfactory career outcomes [16]. Moreover, career adaptability is also considered a psychological capital that is assumed to support employees’ career-oriented behavior to attain career growth and success [39]. Individuals with high career adaptability are able to change their mindset quickly, identify their strengths and interests, and seek out valuable suggestions, which plays an important role in achieving subjective career success [40]. Therefore, career adaptability has a positive impact on subjective career success.

To sum up, individuals with a proactive career orientation actively plan and manage their careers, and they are more inclined to seek opportunities for developing themselves, collect more resources, and become more competitive, thereby improving their career adaptability [41]. Successful adaptation, in turn, has a positive impact on their subjective career success by involving them in proactive behavior toward achieving their career goals and enhancing meaningfulness in their work [42]. In this regard, career adaptability may play a mediating role between proactive career orientation and subjective career success. Previous studies have demonstrated that career adaptability is a key mediator of the career development process based on career construction theory. For example, Nilforooshan and Salimi’s (2016) study found that career adaptability dimensions play a mediating role between specific personality dimensions (i.e., activity, neuroticism, and sensation seeking) and career engagement [43]. Chui et al. (2022) also argue that career adaptability mediates the effect of protean career orientation on career optimism [44]. Taken together, proactive career orientation enhances employees’ career adaptability, leading to greater subjective career success. We propose that:

**Hypothesis 2.**  *Career adaptability mediates the relationship between proactive career orientation and subjective career success*.

### 2.3. Moderating Role of Mentoring

As career construction theory describes, subjective adaptation willingness predicts career adaptability, which in turn leads to adaptive behaviors and outcomes, all of these processes are influenced by the situation [16]. As an important contextual resource, mentoring plays a critical role in one’s career development by providing employees with information, access to social networks, and various support [45]. Those who have been appropriately mentored will report greater career advancement than those who have not [24]. That is, mentoring may act as a moderator in the mechanism by which proactive career orientation influences subjective career success.

Mentoring produces developmental benefits linked to the mentee’s work and career [23]. Particularly, a mentor who has more experience can share more effective information and guidance with his or her mentees through training or daily communication, hence reducing mentees’ cost of trying [46]. Additionally, employees with proactive career orientation actively seek out various opportunities to develop themselves, and the mentor can provide them with skill-related coaching, challenging tasks, feedback, and suggestions for their professional growth [47]. Employees who have received mentoring are more likely to develop a personal career identity and perform self-regulatory behaviors [48]. If employees get encounter a setback at work, the mentor will also provide them with psychological support, such as acceptance, friendship, and counseling [49], steering mentees out of trouble as soon as possible to reengage with their work with a more positive attitude. Employees who receive appropriate mentoring are better able to control their psychological and emotional states and successfully cope with challenges in their careers, which contributes to career adaptability [24]. We hypothesize that:

**Hypothesis 3.**  *Mentoring moderates the relationship between proactive career orientation and career adaptability, such that the positive relationship is stronger for those who receive more mentoring than less mentoring*.

Previous research demonstrates that mentoring is a valuable resource in the workplace because of its ability to foster positive individual outcomes, such as subjective career success [50]. When mentees are able to adapt to their careers, the mentor can provide more effective guidance, such as providing mentees with promotion strategies, learning opportunities, and helping them solve complex problems at work, which contribute to improving mentees’ job satisfaction [51]. Furthermore, the mentor has abundant resources and can provide various career support for mentees, such as the opportunity for mentees to present themself to senior leaders and internal information about the organization. The resources help mentees with strong adaptability to be hopeful about their career development, thus improving their career satisfaction [52]. Moreover, individuals with career adaptability often encounter confusion in their career development. In this regard, the mentor not only provides them with timely feedback and correction and targeted training for their deficiencies but also helps them to adjust their career planning according to their own conditions, leading to subjective career success [53]. Moreover, as a role model for mentees, the mentor provides mentees with the rules that govern effective behavior in the organization [54], which influences and motivates the mentees to facilitate their subjective career success. Therefore, we present the following hypothesis:

**Hypothesis 4.**  *Mentoring moderates the relationship between career adaptability and subjective career success, such that the positive relationship is stronger for those who receive more mentoring than less mentoring*.

Overall, mentoring provides vocational support, social support, and role model support for mentees in their career development [55], allowing mentees to adapt to the organization, work, and job roles as soon as possible. Then, the mentor provides resources, support, and feedback to the mentees regarding career adaptability, helping them to obtain professional skills, self-esteem, and belongingness to achieve subjective career success. Integrating the mediated (H2) and moderated (H3 and H4) relationships developed in the study, we propose a two-stage moderated mediation model, such that the indirect effects of proactive career orientation on subjective career success via career adaptability will be contingent upon mentoring. More specifically, mentoring is likely to strengthen the relationship between proactive career orientation and career adaptability (first-stage moderation), as well as the relationship between career adaptability and subjective career success (second-stage moderation). Therefore, we propose that:

**Hypothesis 5.**  *Mentoring moderates the indirect effect of proactive career orientation on subjective career success via career adaptability, such that both the path between career orientation and career adaptability and the path between career adaptability and subjective career success are stronger when mentoring is more rather than less.*

## 3. Research Methods

### 3.1. Sample and Procedure

To evaluate the proposed model described in this study, data were collected through a questionnaire survey that targeted specific Chinese industries, including manufacturing, education, information systems, tourism, and services. To enhance the validity of the sample size, a snowball sampling technique was employed, considering its suitability within the Chinese context. Initially, we leveraged the personal connections of our Master of Business Administration (MBA) and Doctor of Business Administration (DBA) students to establish contact with Human Resource (HR) directors in potential organizations. Subsequently, our research assistants utilized various communication channels, such as communication software (WeChat, QQ), as well as email, to provide a comprehensive explanation of the research objectives. Upon obtaining informed consent to participate, research assistants distributed the questionnaires to the respective organizations and collaborated with their HR departments to facilitate the collection of data. The researcher ensured participants’ privacy by informing them that the questionnaire would be completed anonymously and only required the last four digits of their personal mobile phone numbers as matching codes. Furthermore, to mitigate common method bias, a three-stage survey was employed for data collection, with a one-month interval between each stage. The survey spanned approximately three months. A total of 350 questionnaires were distributed in this study and 300 were returned, with a return rate of 85.7%. Among them, 4 questionnaires had matching problems and were excluded from the analysis. As a result, a total of 296 valid questionnaires were used for data analysis, resulting in a valid response rate of 84.6%. Out of the 296 valid questionnaires used in this study, 131 respondents (44.3%) identified as male, while 165 respondents (55.7%) identified as female. In terms of marital status, 64 respondents (21.6%) reported being married, while 232 respondents (78.4%) reported being unmarried. Furthermore, the majority of respondents, 263 individuals (88.9%), reported having attained at least a college degree or higher education. The average age of respondents was 26.30 years old (SD = 8.49), and the average job tenure was 4.85 years (SD = 7.36).

### 3.2. Measures

In this study, the variables that were analyzed were based on the established scales that have been previously developed and validated in the research literature. To ensure the validity of these measures in the Chinese context, a back-to-back translation procedure was utilized for measures that were initially developed in English. The study utilized a five-point Likert-type scale to measure all variables, with ratings ranging from 1 (strongly disagree) to 5 (strongly agree).

Proactive career orientation: We utilized a 22-item, three-dimensional scale developed by Briscoe et al. (2006) to assess proactive career orientation [30]. The scale consisted of items such as “I am accountable for the success or failure of my career” (for self-directed, with eight items), “The evaluation of others regarding my career choices is of little concern to me” (for values-driven, with six items), and “I actively seek job opportunities that facilitate learning and growth” (for psychological mobility, with eight items). The Cronbach’s alpha for this scale was 0.96.

Career adaptability: To assess career adaptability, we employed a 24-item, four-dimensional scale developed by Hou et al. (2012) [56]. The scale comprised items such as “I frequently contemplate what my future holds” (for concern, with six items), “I maintain a positive outlook even during challenging times” (for control, with six items), “I enjoy exploring new environments and ideas” (for curiosity, with six items), and “I am skilled at completing tasks effectively and efficiently” (for confidence, with six items). The Cronbach’s alpha for this scale was 0.98.

Mentoring: Mentoring was assessed using a 15-item, three-dimensional scale developed by Scandura and Ragins (1993) [57]. The scale consisted of items such as “My mentor shows a genuine interest in my career development” (for career coaching, with six items), “I share personal information and receive emotional support from my mentor” (for psycho-social support, with five items), and “I aspire to emulate my mentor’s behavior and values” (for role modeling, with four items). The Cronbach’s alpha for this scale was 0.98.

Subjective career success: We employed a 5-item scale developed by Greenhaus et al. (1990) to measure subjective career success [58]. A sample item was: “I am content with the level of success I have attained in my career”. The Cronbach’s alpha for this scale was 0.86.

Control variables: To account for potential confounding effects, we included gender, age, and tenure as control variables in our analyses. We included gender as a control variable due to its potential influence on both career adaptability [59] and subjective career success [60]. Age was also controlled for, as it has been found to positively predict both career adaptability and career success [8,61]. Finally, we included tenure as a control variable based on the recommendation of Judge et al. (1995), who argued that job tenure is related to subjective career success [62].

## 4. Results

### 4.1. Confirmatory Factor Analysis

We assessed the discriminant validity of all variables using Mplus 8.3 (Muthén and Muthén, 2017) by conducting a series of confirmatory factor analyses (CFA) in our study [63]. The results of the CFA are presented in Table 1. As indicated, the hypothesized four-factor model, comprising proactive career orientation, career adaptability, mentoring, and subjective career success, fit the data well (χ2 = 3005.48, df = 2073, CFI = 0.95, TLI = 0.9, SRMR = 0.05, RMSEA = 0.04). Additionally, the comparison of the hypothesized model with all alternative models through chi-square difference tests demonstrated that our hypothesized model provided the best fit to the data, supporting the discriminant validity of the measures.

### 4.2. Descriptive Statistics and Correlations

In this study, descriptive statistics and correlations were performed using SPSS. To evaluate the potential impact of common method variance (CMV) on our research findings, we conducted Harman’s single factor test, as suggested by Podsakoff et al. (2003) [64]. The test results revealed that the first principal component variance contribution rate was 36.56%, which was lower than the critical value of 50%. This finding suggests that common method variance is not a major issue in our study, and thus, we are able to interpret our results with greater confidence. The means, standard deviations, and correlation coefficients of the variables in Table 2 indicate that proactive career orientation was significantly and positively related to subjective career success (r = 0.42, *p* < 0.001), providing preliminary support for H1. Additionally, proactive career orientation was positively associated with career adaptability (r = 0.41, *p* < 0.001), and career adaptability was positively correlated with subjective career success (r = 0.42, *p* < 0.001). These results satisfy the necessary conditions for examining the mediation.

### 4.3. Hypothesis Testing

We employed the PROCESS tool for SPSS (Hayes, 2012) to evaluate our hypotheses [65]. We examined the mediation hypothesis (H2) using Baron and Kenny’s (1986) approach [66]. First, as displayed in Table 3 Model 1, proactive career orientation was positively associated with subjective career success (b = 0.45, *p* < 0.001), which supports H1. Second, proactive career orientation was positively related to career adaptability (b = 0.45, *p* < 0.001, Model 2), and career adaptability was positively related to subjective career success (b = 0.37, *p* < 0.001, Model 3), meeting the prerequisites for testing the mediation. Third, we added proactive career orientation and career adaptability in the regression model simultaneously (see Table 3 Model 4). The relationship between career adaptability and subjective career success remained significant (b = 0.27, *p* < 0.001), and the regression coefficient of proactive career orientation decreased (from b = 0.45, *p* < 0.001 to b = 0.33, *p* < 0.001), indicating partial mediation. Moreover, we applied the Sobel’s test and the bootstrapping method, resampling 5000 times, to examine the indirect effect of career adaptability between proactive career orientation and subjective career success. The results showed a significant indirect effect of proactive career orientation on subjective career success via career adaptability (0.12, 95% CI of [0.06, 0.20], Z = 4.13, *p* < 0.001), which did not include zero, thus supporting H2.

**Figure 2:**
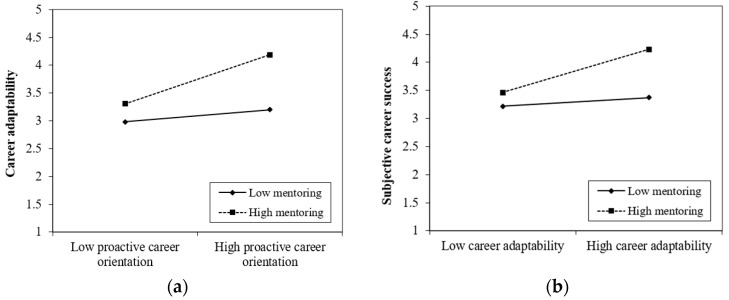
The moderating role of mentoring: (**a**) the moderating role of mentoring on the relationship between proactive career orientation and career adaptability; (**b**) the moderating role of mentoring on the relationship between career adaptability and subjective career success.

Hypotheses 3 and 4 proposed that mentoring would moderate the relationships between proactive career orientation and career adaptability, and between career adaptability and subjective career success. The results from the moderation model, presented in Table 4, support these hypotheses by revealing a significant interaction effect between proactive career orientation and mentoring on career adaptability (B = 0.24, SE = 0.05, *p* < 0.001), and between career adaptability and mentoring on subjective career success (B = 0.18, SE = 0.04, *p* < 0.001). To illustrate the interaction effect across different levels of the moderator (mean ± 1 SD), we have depicted these two interactions in Figure 2. As expected, Figure 2a demonstrates that the positive association between proactive career orientation and career adaptability is stronger when there is more mentoring. Similarly, Figure 2b shows that the positive relationship between career adaptability and subjective career success is stronger with higher levels of mentoring.

To examine the moderated mediation model (H5), we tested the conditional indirect effect of proactive career orientation on subjective career success through career adaptability at three different levels of mentoring (one standard deviation above the mean, the mean, and one standard deviation below the mean). As presented in Table 4, the indirect effect was found to be insignificant (effect = 0.01, 95% CI [−0.01, 0.06]) for lower levels of mentoring but significant for higher levels of mentoring (effect = 0.18, 95% CI [0.06, 0.37]). Furthermore, the confidence interval of the difference between higher and lower levels of mentoring was [0.04, 0.35], indicating a significant moderated mediation effect. These results provide support for H5.

## 5. Discussion

Drawing on career construction theory, our study explores the relationship between proactive career orientation and subjective career success, taking into account the mediating role of career adaptability and the moderating role of mentoring. Our results demonstrate a positive relationship between proactive career orientation and subjective career success. Additionally, we find that career adaptability partially mediates the relationship between proactive career orientation and subjective career success. Furthermore, mentoring has a positive moderating effect on the relationship between proactive career orientation and career adaptability, the relationship between career adaptability and subjective career success, and the indirect effect of proactive career orientation on subjective career success through career adaptability.

### 5.1. Theoretical Contributions

First, based on career construction theory, we examined the relationship between proactive career orientation and subjective career success. Although recent research has devoted much attention to subjective career success, researchers have rarely explored how contemporary career attitudes (e.g., proactive career orientation) affect career-related outcomes [10]. In line with the existing literature that highlights the influence of proactive career orientation [14,15], this study finds that individuals with proactive career orientation are more likely to obtain subjective career success. We also consider proactive career orientation as “adaptive readiness“ to extend the application of career construction theory.

In addition, we find that career adaptability mediates the relationship between proactive career orientation and subjective career success. Consistent with previous research, we demonstrated that career adaptability plays a key role in subjective career success in individual career development [8,67]. Our findings not only support career construction theory but also reveal the internal processes induced by proactive career orientation that explain subjective career success.

Third, we clarify that mentoring as a boundary condition strengthens the mechanism whereby proactive career orientation influences subjective career success. Given that career adaptability is considered a critical resource for shaping adaptive strategies and actions aimed at achieving adaptive goals [16], we reveal the effectiveness of mentoring in protecting and developing employees’ career adaptability. This finding is in line with previous studies to consolidate that mentoring plays an important role in mentee’s career outcomes [68,69,70]. Additionally, we extend career construction theory by showing that mentoring as a situational factor interacts with individual career orientation to influence individual career constructs, answering the call for an integration of interaction effects in understanding career development [71].

### 5.2. Practical Implications

There are two practical implications of this study. First, our study confirmed that proactive career orientation is positively related to subjective career success through career adaptability. This suggests that the career planning centers at universities could benefit from interventions to enhance students’ career proactivity [14]. For instance, universities can provide students with career training courses to assist them in setting specific career goals that are aligned with their interests and values. Universities and companies then cooperate to establish an internship platform with feedback so that students can gradually establish their career orientation as they learn knowledge and engage in practice, helping them improve their career adaptability and subjective career success after entering the workforce. In addition, it is necessary for employees to shift from the traditional career attitude of reliance on the organization to a proactive career orientation in order to improve career adaptability and attain subjective career success. As for managers, when they assign jobs and tasks to employees, they should pay attention not only to the employees’ own abilities but also to their career orientation. Individuals with a proactive career orientation take charge of their careers based on their interests and values, so managers need to ensure that organizational goals are compatible with employees’ goals as much as possible, especially in an open and flexible organizational structure. Under such circumstances, employees can be motivated to perform their jobs more effectively.

Second, mentoring strengthens the process whereby proactive career orientation influences subjective career success. We emphasize career adaptability, as a malleable construct, can be developed by the interaction of mentoring and proactive career orientation, ultimately contributing to subjective career success. Therefore, to maximize the benefits of mentoring, we recommend that organizations value and invest in a well-designed mentorship program, which would effectively foster resource acquisition by employees and facilitate their adaptation to the unpredictable and challenging work environment. In this program, managers should focus on employee’s career interests and career development needs and match them with the organization’s goals Mentors should provide personalized guidance and training to mentees, improve their career adaptability, and face the changing environment together with members of the organization, which helps to improve the employment efficiency of the company. Mentoring not only improves the competitiveness and attractiveness of an organization in a dynamic and flexible work environment but also helps employees obtain subjective career success and become more willing to create value for the organization, which helps build a win–win relationship between employees and the organization.

### 5.3. Limitations and Future Directions

There are several limitations to this study that should be acknowledged. First, the data source used in our study is singular and does not track the long-term development of employees’ subjective career success. Therefore, the interpretation of causality is somewhat limited. To overcome this limitation, future research could adopt a longitudinal study design to track long-term changes in subjective career success and explore possible predictors.

Second, due to time and cost constraints, our sample size was small, which restricts the generalizability of the findings. Future studies should expand the sample size to enhance the external validity of the results. Moreover, this study did not distinguish between specific occupations, and different occupations may yield different research findings due to varying work characteristics and work environments. Future research could investigate specific occupations, such as technology-based employees or college teachers, to study the impact of proactive career tendencies on subjective career success.

Third, this study only explored the moderating role of mentoring. However, career construction theory suggests that many situational factors can influence the whole process of career construction [6]. Therefore, future research could explore the influence of other situational factors on the mechanism of individual proactive career orientation on subjective career success through career adaptability, such as social support or personal–organizational fit. In addition, situational factors at the team or organizational level are also worthy of attention, such as climate, human resource policies, or practices, etc.

## 6. Conclusions

By clarifying the relationships between proactive career orientation and subjective career success, this study contributes to the advancement of theory and research on career development. Our findings, grounded in career construction theory, highlight the impact of proactive career orientation on subjective career success through career adaptability, with mentoring moderating the process. Despite the limitations of this study, we hope that our research will encourage other scholars to deepen their understanding of the fields of individual career development.

## Figures and Tables

**Table 1:** The results of confirmatory factor analyses.

Measurement Model	χ^2^	df	Δχ^2^	CFI	TLI	SRMR	RMSEA
The hypothesized four-factor model	3005.48	2073		0.95	0.95	0.05	0.04
Three-factor model (combining CA and SCS)	3563.87	2076	558.39 ***	0.91	0.91	0.07	0.05
Two-factor model (combining CA, MT, and SCS)	7776.38	2078	4770.90 ***	0.67	0.66	0.13	0.10
One-factor model (combining PCO, CA, MT, and SCS)	11,042.64	2079	8037.16 ***	0.49	0.47	0.19	0.12

Note: *N* = 296; *** *p* < 0.001; CFI = comparative fit index; TLI = Tucker–Lewis index; SRMR = standardized root mean square residual; RMSEA = root mean square of approximation; PCO = proactive career orientation; CA = career adaptability; MT = mentoring; SCS = subjective career success.

**Table 2:** Means, standard deviations, and correlations.

Variables	Mean	SD	1	2	3	4	5	6
1. Gender	0.56	0.50						
2. Age	26.3	8.49	−0.29 ***					
3. Tenure	4.85	7.36	−0.23 ***	0.75 ***				
4. Proactive career orientation	3.68	0.65	0.20 ***	0.03	−0.04			
5. Career adaptability	3.78	0.80	0.24 ***	0.02	−0.04	0.41 ***		
6. Mentoring	3.50	1.06	0.09	−0.07	−0.11	0.09	0.43 ***	
7. Subjective career success	3.71	0.73	0.20 **	−0.13 **	−0.18 **	0.42 ***	0.42 ***	0.48 ***

Note: *N* = 296; SD: standard deviations; ** *p* < 0.01; *** *p* < 0.001.

**Table 3:** Regression results for direct effect model and mediation model.

Variables	Model 1 X→Y	Model 2 X→M	Model 3 M→Y	Model 4 X→M→Y
Constant	2.10	1.72	2.39	1.64
Gender	0.11	0.28 **	0.09	0.04
Age	−0.00	0.01	−0.00	−0.01
Tenure	−0.01	−0.01	−0.01	−0.01
Proactive career orientation	0.45 ***	0.45 ***		0.33 ***
Career adaptability			0.37 ***	0.27 ***
R^2^	0.21	0.20	0.21	0.28
F	19.34 ***	17.72 ***	18.94 ***	22.31 ***
Indirect effect				
	Value	SE	z	*p*
Sobel	0.12	0.03	4.13	<0.001
Bootstrap results for indirect effect				
	M	SE	LLCI	ULCI
Effect	0.12	0.04	0.06	0.20

Note: *N* = 296; ** *p* < 0.01; *** *p* < 0.001; Unstandardized regression coefficients are reported; Bootstrap sample size 5000; LL = lower limit; UL = upper limit; CI = confidence interval.

**Table 4:** Regression results for conditional indirect.

Predictor	Career Adaptability		Subjective Career Success	
	B	SE	B	SE
Moderation model				
Constant	3.42	0.17	3.57 ***	0.17
Gender	0.21 **	0.08	0.13 *	0.08
Age	0.01	0.01	0.00	0.01
Tenure	−0.01	0.01	−0.01	0.01
Proactive career orientation	0.42 ***	0.06		
Career adaptability			0.29 ***	0.05
Mentoring	0.31 ***	0.04	0.26 ***	0.04
Proactive career orientation × Mentoring	0.24 ***	0.05		
Career adaptability × Mentoring			0.18 ***	0.04
Moderated mediation model				
	Effect	BootSE	BootLLCI	BootULCI
Low mentoring (Mean − 1 SD)	0.01	0.02	−0.01	0.06
Mentoring (Mean)	0.07	0.03	0.01	0.12
High mentoring (Mean + 1 SD)	0.18	0.08	0.06	0.37
Difference	0.17	0.08	0.04	0.35

Note: *N* = 296; * *p* < 0.05; ** *p* < 0.01; *** *p* < 0.001; Unstandardized regression coefficients are reported; Bootstrap sample size 5000; LL = lower limit; UL = upper limit; CI = confidence interval.

## Data Availability

The data shown in this research are available on request from the corresponding author.
